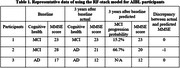# Developing a machine learning stack model to forecast the progression of mild cognitive impairment to Alzheimer’s dementia, using the Australian Imaging, Biomarker & Lifestyle (AIBL) Study dataset

**DOI:** 10.1002/alz.084645

**Published:** 2025-01-03

**Authors:** Chenyin Chu, Yihan Wang, Paul Maruff, Colin L. Masters, Benjamin Goudey, Liang Jin, Yijun Pan, The AIBL research group

**Affiliations:** ^1^ University of Melbourne, Parkville, VIC Australia; ^2^ Florey Institute of Neuroscience and Mental Health, Parkville, VIC Australia; ^3^ Florey Institute of Neuroscience and Mental Health, University of Melbourne, Parkville, VIC Australia; ^4^ Monash University, Parkville, VIC Australia; ^5^ Tohoku University, Sendai Japan

## Abstract

**Background:**

Alzheimer’s disease (AD) is a progressive neurodegenerative condition, with considerable variation in disease progression from the mild cognitive impairment (MCI) stage. Predicting disease progression will support prognostic decisions and patient management. Here we designed a machine learning (ML) stack model, where a classifier was used to differentiate MCI progressors from non‐progressors (i.e. whether an individual with MCI progress to AD), and different regression models were then applied to each category to forecast their Mini‐Mental State Examination (MMSE) score after 3 years.

**Method:**

Demographic and neuropsychological data from 2428 participants of the AIBL study was used for model construction and validation. The model was validated via a stratified 3‐fold cross‐validation approach. Support vector machine (SVM), gradian boosting (GB), random forest (RF) was experimented, and their performance were compared. MMSE score was a predicted variable indicating cognition.

**Result:**

The performance of RF‐stack model was superior to SVM‐ and GB‐stack models. Largest area under the receiver operating characteristic curve (0.85) when RF‐stack model was employed (compared with SVM 0.79 and GB 0.70), signifying the highest accuracy in classify MCI progressors and non‐progressors. RF‐stack model achieved the best performance in predicting MMSE score after 3 years), evidenced by the smallest median mean average error (MAE) (RF 1.51, SVM 1.52, GB 2.51). The MAE indicated how much the predicted MMSE score deviated from the AIBL record. Representative prediction results from RF‐stack model for AIBL participants were presented in Table 1.

**Conclusion:**

Our RF‐stack model relies on neuropsychological test scores and demographic data to predict the probability of an individual progressing from MCI to AD and accurately forecast the MMSE score after 3 years. This model achieved is comparable if not better prediction accuracy than the existing ML models using neuroimaging data. Further model optimization and validation using data collected from other longitudinal AD cohort studies are required.